# Supplementary material for: Evaluation of COVID-19 vaccination strategies with a delayed second dose
Source: PLoS Biol. 2021 Apr 21;19(4):e3001211. doi: 10.1371/journal.pbio.3001211 (PMC8092656; doi:10.1371/journal.pbio.3001211)
Supplement: S1 Text — (DOCX) [file pbio.3001211.s013.docx]

**Supporting file**

**Evaluation of COVID-19 vaccination strategies with a delayed second dose**

Seyed M. Moghadas,^1^ Thomas N Vilches,^2^ Kevin Zhang,^3^ Shokoofeh Nourbakhsh,^1^ Pratha Sah,^4^ Meagan C. Fitzpatrick,^4,5^ Alison P. Galvani^4^

^1^ Agent-Based Modelling Laboratory, York University, Toronto, Ontario, M3J 1P3 Canada

^2^ Institute of Mathematics, Statistics and Scientific Computing, University of Campinas, Campinas SP, Brazil

^3^ Faculty of Medicine, University of Toronto, Toronto, Ontario, M5S 1A8 Canada

^4^ Center for Vaccine Development and Global Health, University of Maryland School of Medicine, 685 W Baltimore St, Baltimore, MD 21201 USA

^5^ Center for Infectious Disease Modeling and Analysis (CIDMA), Yale School of Public Health, New Haven, Connecticut, USA

This appendix provides further details of model parameterization, and additional results for comparing a DSD strategy with the recommended schedule of vaccination for both Pfizer-BioNTech and Moderna vaccines, corresponding to the efficacy of vaccines against infection.

**Table A.** Mixing patterns and the daily number of contacts derived from empirical observations. Daily numbers of contacts were sampled from negative binomial distributions for different scenarios.

| **Age group** | **Proportion of contacts between age groups** | | | | | **No. of daily contacts without self-isolation**  **Mean (SD)** | **No. of daily contacts for self-isolated individuals**  **Mean (SD)** |
| --- | --- | --- | --- | --- | --- | --- | --- |
|  | **0-4** | **5-19** | **20-49** | **50-65** | **65+** |  |  |
| 0-4 | 0.2287 | 0.1839 | 0.4219 | 0.1116 | 0.0539 | 10.21 (7.65) | 2.86 (2.14) |
| 5-19 | 0.0276 | 0.5964 | 0.2878 | 0.0591 | 0.0291 | 16.793 (11.7201) | 4.70 (3.28) |
| 20-49 | 0.0376 | 0.1454 | 0.6253 | 0.1423 | 0.0494 | 13.795 (10.5045) | 3.86 (2.95) |
| 50-65 | 0.0242 | 0.1094 | 0.4867 | 0.2723 | 0.1074 | 11.2669 (9.5935) | 3.15 (2.66) |
| 65+ | 0.0207 | 0.1083 | 0.4071 | 0.2193 | 0.2446 | 8.0027 (6.9638) | 2.24 (1.95) |

**Table B.** Description of model parameters and their estimates.

| **Description** | | **0–4** | **5–19** | **20–49** | **50–64** | **65–79** | **80+** | **Source** |
| --- | --- | --- | --- | --- | --- | --- | --- | --- |
| Transmission probability per contact during presymptomatic stage | | Depending on the level of (herd immunity)  0.042 (10%), 0.0465 (20%), 0.054 (30%) | | | | | | Calibrated to  R=1.2 [1] |
| Incubation period (days) | | LogNormal(shape: 1.434, scale: 0.661) | | | | | | [2] |
| Asymptomatic period (days) | | Gamma(shape: 5, scale: 1) | | | | | | Derived from  [3,4] |
| Presymptomatic period (days) | | Gamma(shape: 1.058, scale: 2.174) | | | | | | Derived from  [5,6] |
| Infectious period from onset of symptoms (days) | | Gamma(shape: 2.768, scale: 1.1563) | | | | | | Derived from  [3] |
| Proportion of infections that are asymptomatic | | 0.30 | 0.38 | 0.33 | 0.33 | 0.19 | 0.19 | [7-9] |
| Proportion of symptomatic cases that exhibit mild symptoms | | 0.95 | 0.90 | 0.85 | 0.60 | 0.20 | 0.20 | [10,11] |
| Proportion of cases hospitalized with one or more comorbidities | | 37.6% | | | | | | [12,13] |
|  | Non-ICU | 67% | | | | | |  |
|  | ICU | 33% | | | | | |  |
| Proportion of cases hospitalized without any comorbidities | | 9% | | | | | | [12,13] |
|  | Non-ICU | 75% | | | | | |  |
|  | ICU | 25% | | | | | |  |
| Length of non-ICU stay (days) | | Gamma(shape: 4.5, scale: 2.75) | | | | | | Derived from  [14,15] |
| Length of ICU stay  (days) | | Gamma(shape: 4.5, scale: 2.75) + 2 | | | | | | Derived from  [14,15] |

**Table C.** Vaccination coverage of different age groups over a one-year time horizon.

| **Age group** | **0-17** | **18-49** | **50-64** | **65-79** | **80+** |
| --- | --- | --- | --- | --- | --- |
| Vaccination coverage | 0% | 59% | 63% | 94% | 96% |

**Results with vaccine efficacy set at upper (Fig A) and lower (Fig B) bounds of the estimated ranges without waning of the first-dose efficacy.**

**
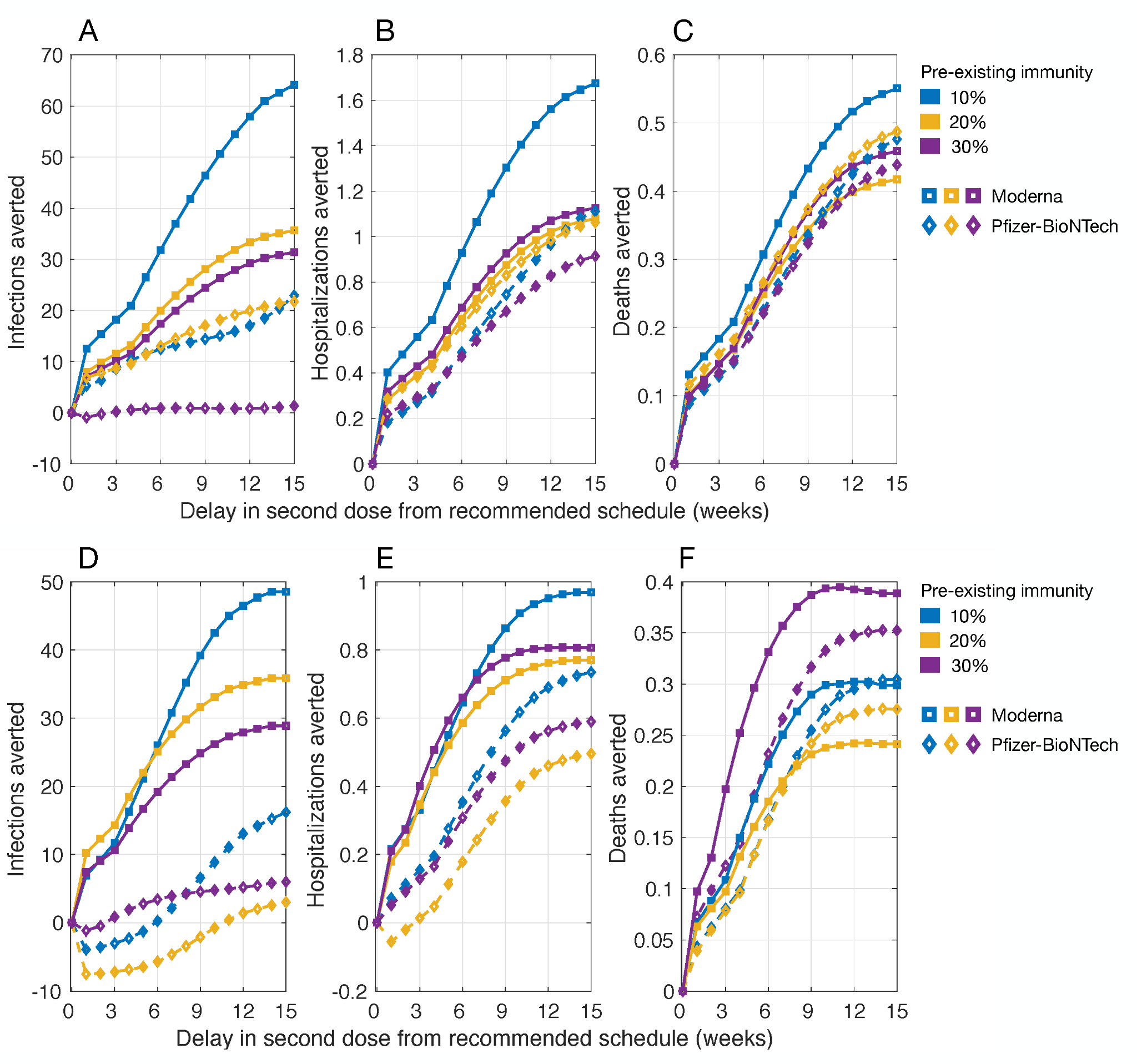
**

**Figure A.** Projected reduction of infections, hospitalizations, and deaths for a DSD vaccination program compared to the recommended schedule of two-doses of Pfizer-BioNTech (with a 21-day interval) and Moderna (with a 28-day interval) vaccines. The daily vaccination rate was (A,B,C) 30 doses and (D,E,F) 45 doses per 10,000 population. Vaccine efficacy was set to the upper bound of estimated ranges (Fig 1 in Main Text) without waning of first-dose efficacy prior to the administration of the second dose. The individual numerical values for A-C and D-F are listed in S5 Data and S6 Data, respectively.

**
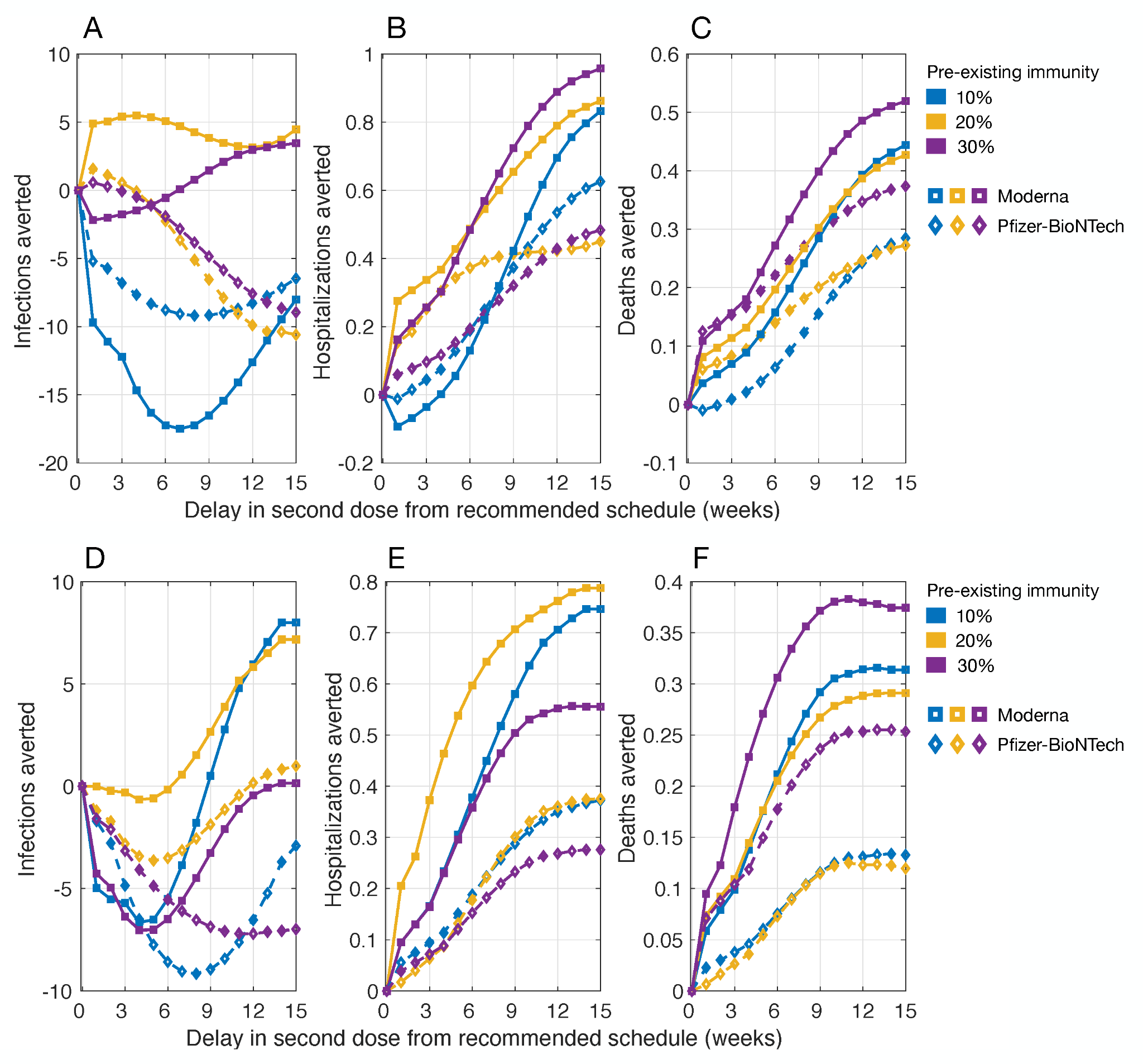
**

**Figure B.** Projected reduction of infections, hospitalizations, and deaths for a DSD vaccination program compared to the recommended schedule of two-doses of Pfizer-BioNTech (with a 21-day interval) and Moderna (with a 28-day interval) vaccines. The daily vaccination rate was (A,B,C) 30 doses and (D,E,F) 45 doses per 10,000 population. Vaccine efficacy was set to the lower bound of estimated ranges (Fig 1 in Main Text) without waning of first-dose efficacy prior to the administration of the second dose. The individual numerical values for A-C and D-F are listed in S7 Data and S8 Data, respectively.

**
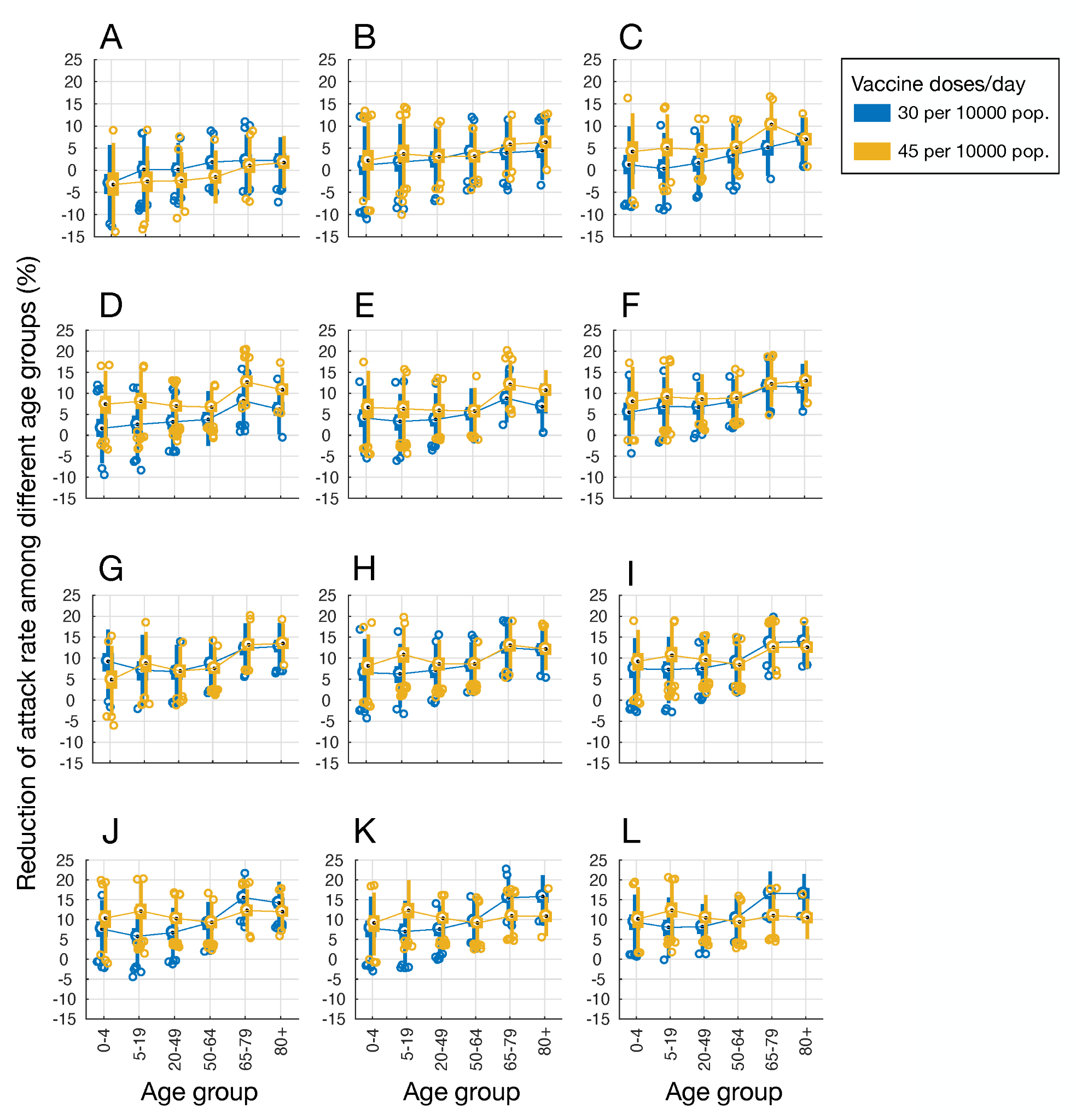
**

**Figure C.** Projected reduction of attack rates among different age groups in a DSD strategy with Moderna vaccines. The level of pre-existing immunity was 20% and vaccine efficacy set at the mean values of estimated ranges. Panels A to L correspond to the delay of 1 to 12 weeks in administering the second dose from the recommended schedule, without waning efficacy of the first-dose. The raw data to generate A-F using a bootstrap method are available at: <https://github.com/thomasvilches/delay_data>.

**
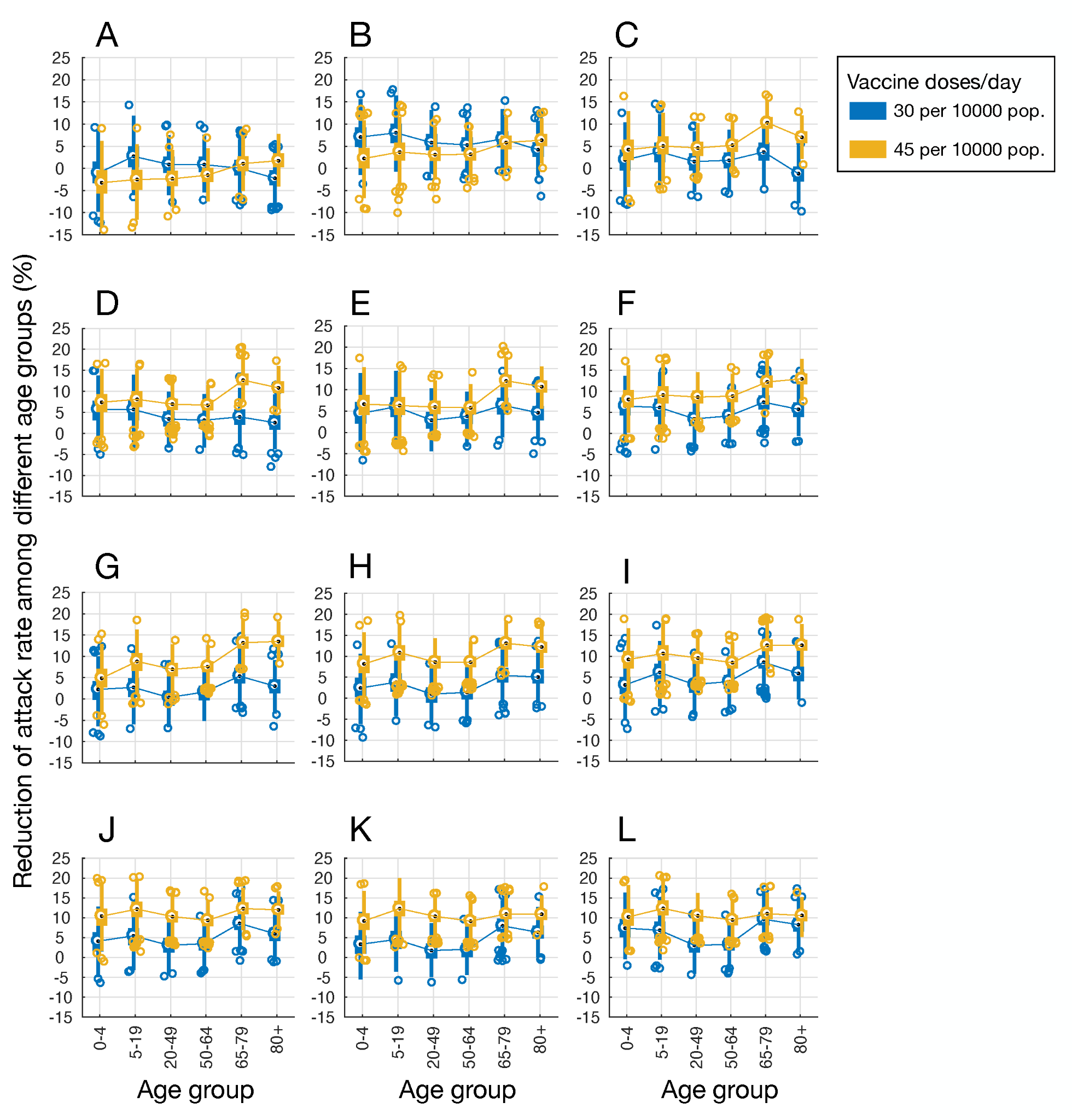
**

**Figure D.** Projected reduction of attack rates among different age groups in a DSD strategy with Pfizer-BioNTech vaccines. The level of pre-existing immunity was 20% and vaccine efficacy set at the mean values of estimated ranges. Panels A to L correspond to the delay of 1 to 12 weeks in administering the second dose from the recommended schedule, without waning efficacy of the first-dose. The raw data to generate A-F using a bootstrap method are available at <https://github.com/thomasvilches/delay_data>.

**Results with vaccine efficacy set at upper (Fig E) and lower (Fig F) bounds of the estimated ranges with waning of the first-dose efficacy.**


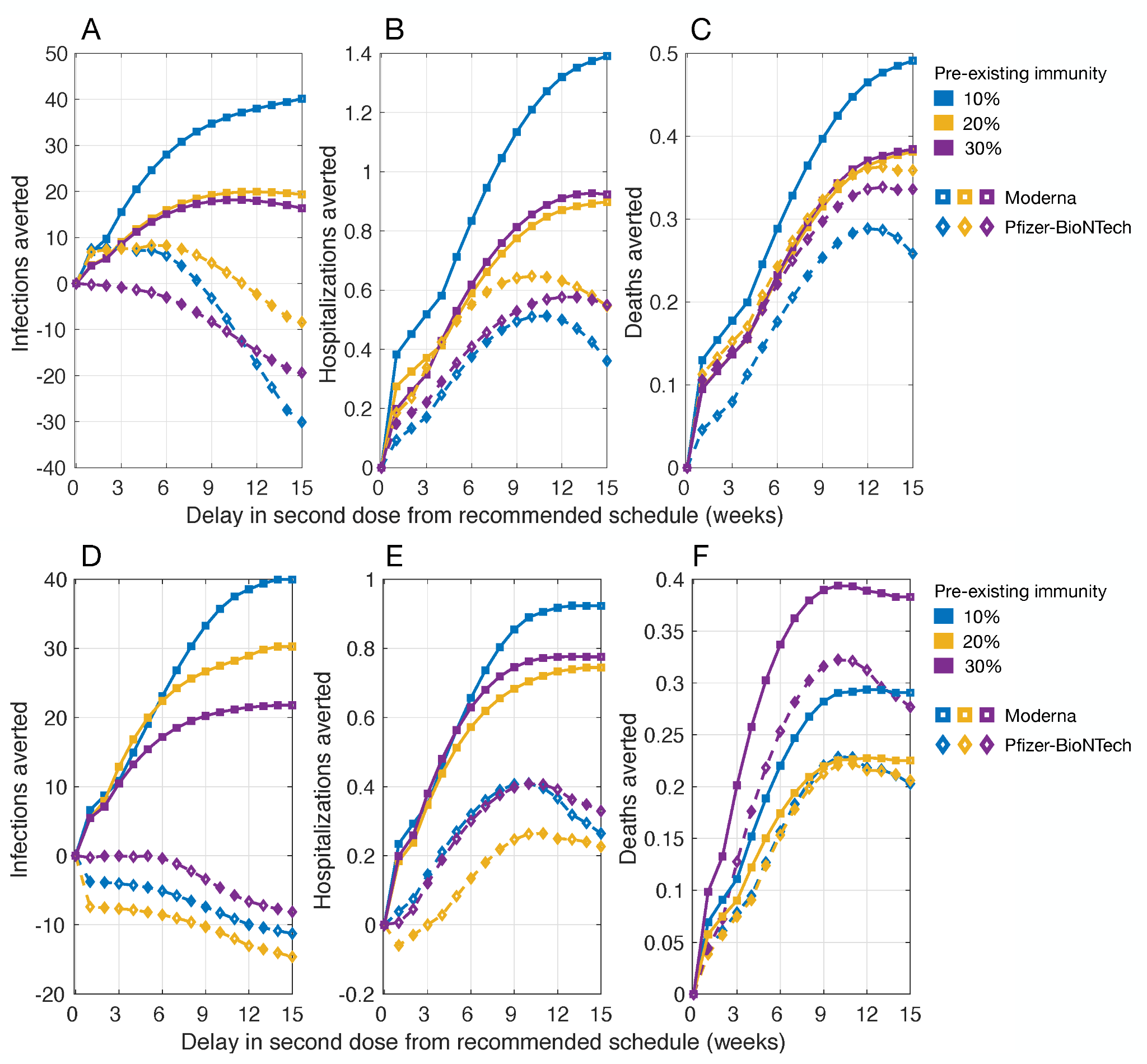


**Figure E.** Projected reduction of infections, hospitalizations, and deaths for a DSD vaccination program compared to the recommended schedule of two-doses of Pfizer-BioNTech (with a 21-day interval) and Moderna (with a 28-day interval) vaccines. The daily vaccination rate was (A,B,C) 30 doses and (D,E,F) 45 doses per 10,000 population. Vaccine efficacy was set to the upper bound of estimated ranges (Fig 1 in Main Text), and the waning rate of first-dose efficacy was 5% per week, starting from week 7 after the first dose prior to the administration of the second dose. The individual numerical values for A-C and D-F are listed in S9 Data and S10 Data, respectively.

**
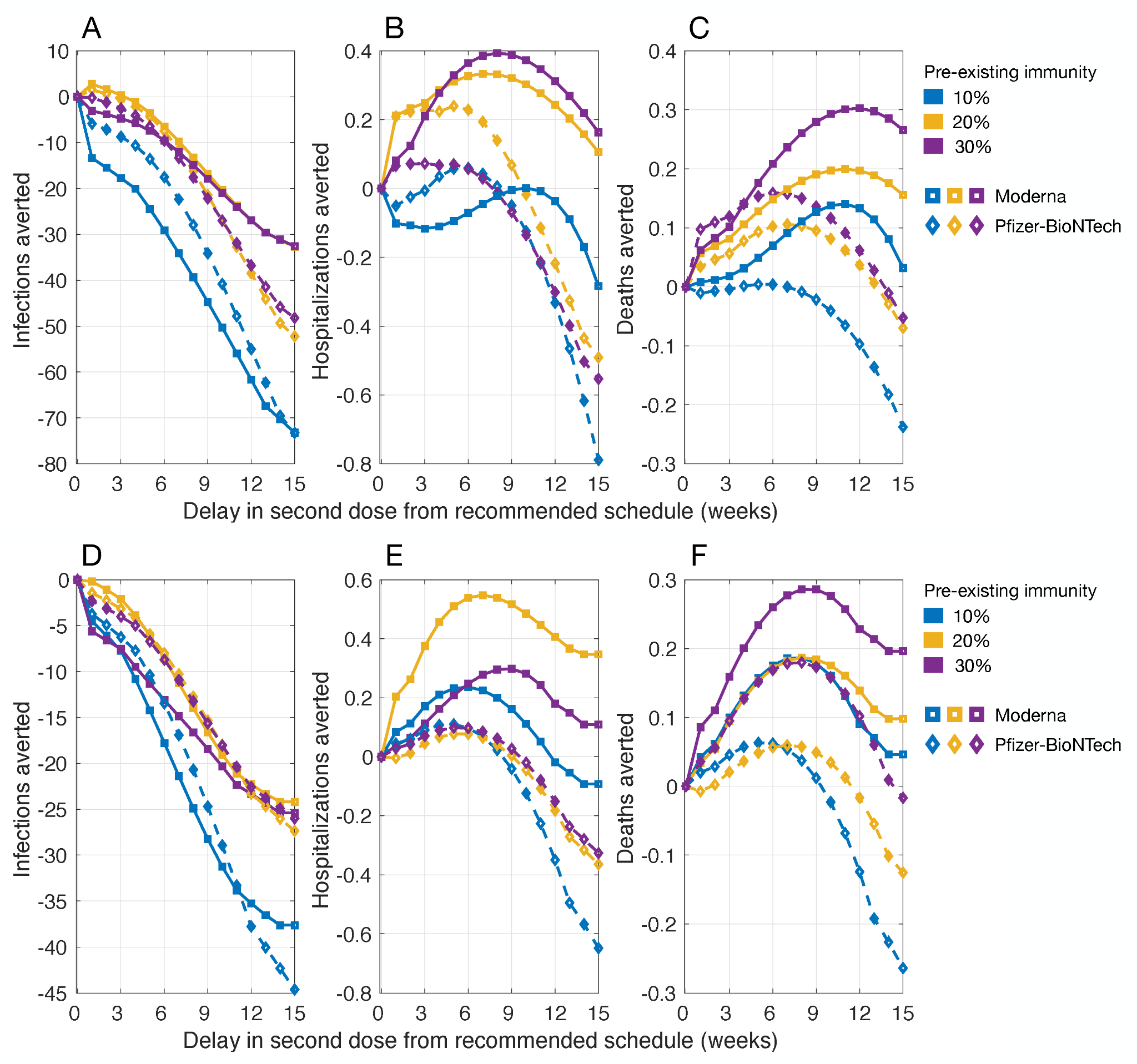
**

**Figure F.** Projected reduction of infections, hospitalizations, and deaths for a DSD vaccination program compared to the recommended schedule of two-doses of Pfizer-BioNTech (with a 21-day interval) and Moderna (with a 28-day interval) vaccines. The daily vaccination rate was (A,B,C) 30 doses and (D,E,F) 45 doses per 10,000 population. Vaccine efficacy was set to the lower bound of estimated ranges (Fig 1 in Main Text), and the waning rate of first-dose efficacy was 5% per week, starting from week 7 after the first dose prior to the administration of the second dose. The individual numerical values for A-C and D-F are listed in S11 Data and S12 Data, respectively.

**References**

1. Systrom K, Vladek T, Krieger M. Rt.live. GitHub repository. 2020. Available: [https://github.com/rtcovidlive/covid-model. Accessed 16 Nov 2020](https://github.com/rtcovidlive/covid-model.%20Accessed%2016%20Nov%202020).
2. Li Q, Guan X, Wu P, Wang X, Zhou L, Tong Y, et al. Early Transmission Dynamics in Wuhan, China, of Novel Coronavirus-Infected Pneumonia. N Engl J Med. 2020;382: 1199–1207. doi:10.1056/NEJMoa2001316
3. Li R, Pei S, Chen B, Song Y, Zhang T, Yang W, et al. Substantial undocumented infection facilitates the rapid dissemination of novel coronavirus (SARS-CoV-2). Science. 2020;368: 489–493. doi:10.1126/science.abb3221
4. Gatto M, Bertuzzo E, Mari L, Miccoli S, Carraro L, Casagrandi R, et al. Spread and dynamics of the COVID-19 epidemic in Italy: Effects of emergency containment measures. Proc Natl Acad Sci. 2020;117: 10484–10491. doi:10.1073/pnas.2004978117
5. He X, Lau EHY, Wu P, Deng X, Wang J, Hao X, et al. Temporal dynamics in viral shedding and transmissibility of COVID-19. Nat Med. 2020;26: 672–675. doi:10.1038/s41591-020-0869-5
6. Moghadas SM, Fitzpatrick MC, Sah P, Pandey A, Shoukat A, Singer BH, et al. The implications of silent transmission for the control of COVID-19 outbreaks. Proc Natl Acad Sci. 2020;117: 17513–17515. doi:10.1073/pnas.2008373117
7. Mizumoto K, Kagaya K, Zarebski A, Chowell G. Estimating the asymptomatic proportion of coronavirus disease 2019 (COVID-19) cases on board the Diamond Princess cruise ship, Yokohama, Japan, 2020. Euro Surveill Bull Eur Sur Mal Transm Eur Commun Dis Bull. 2020;25. doi:10.2807/1560-7917.ES.2020.25.10.2000180
8. Nishiura H, Kobayashi T, Miyama T, Suzuki A, Jung S, Hayashi K, et al. Estimation of the asymptomatic ratio of novel coronavirus infections (COVID-19). Int J Infect Dis. 2020;94: 154–155. doi:10.1016/j.ijid.2020.03.020
9. Kimball A, Hatfield KM, Arons M, James A, Taylor J, Spicer K, et al. Asymptomatic and Presymptomatic SARS-CoV-2 Infections in Residents of a Long-Term Care Skilled Nursing Facility — King County, Washington, March 2020. MMWR Morb Mortal Wkly Rep. 2020;69: 377–381. doi:10.15585/mmwr.mm6913e1
10. Shoukat A, Wells CR, Langley JM, Singer BH, Galvani AP, Moghadas SM. Projecting demand for critical care beds during COVID-19 outbreaks in Canada. CMAJ Can Med Assoc J J Assoc Medicale Can. 2020;192: E489–E496. doi:10.1503/cmaj.200457
11. Moghadas SM, Shoukat A, Fitzpatrick MC, Wells CR, Sah P, Pandey A, et al. Projecting hospital utilization during the COVID-19 outbreaks in the United States. Proc Natl Acad Sci U S A. 2020;117: 9122–9126. doi:10.1073/pnas.2004064117
12. Garg S, Kim L, Whitaker M, O’Halloran A, Cummings C, Holstein R, et al. Hospitalization Rates and Characteristics of Patients Hospitalized with Laboratory-Confirmed Coronavirus Disease 2019 — COVID-NET, 14 States, March 1–30, 2020. MMWR Morb Mortal Wkly Rep. 2020;69: 458–464. doi:10.15585/mmwr.mm6915e3
13. CDC COVID-19 Response Team, CDC COVID-19 Response Team, Chow N, Fleming-Dutra K, Gierke R, Hall A, et al. Preliminary Estimates of the Prevalence of Selected Underlying Health Conditions Among Patients with Coronavirus Disease 2019 — United States, February 12–March 28, 2020. MMWR Morb Mortal Wkly Rep. 2020;69: 382–386. doi:10.15585/mmwr.mm6913e2
14. Yang X, Yu Y, Xu J, Shu H, Xia J, Liu H, et al. Clinical course and outcomes of critically ill patients with SARS-CoV-2 pneumonia in Wuhan, China: a single-centered, retrospective, observational study. Lancet Respir Med. 2020;8: 475–481. doi:10.1016/S2213-2600(20)30079-5
15. Sanche S, Lin YT, Xu C, Romero-Severson E, Hengartner N, Ke R. High Contagiousness and Rapid Spread of Severe Acute Respiratory Syndrome Coronavirus 2. Emerg Infect Dis. 2020;26: 1470–1477. doi:10.3201/eid2607.200282
